# Role of skip N2 lymph node metastasis for patients with the stage III-N2 lung adenocarcinoma: a propensity score matching analysis

**DOI:** 10.1186/s12890-023-02437-0

**Published:** 2023-04-28

**Authors:** Shize Wang, Shaonan Xie, Yaqing Han, Maogang Gao, Xin Su, Qingyi Liu

**Affiliations:** Department of Thoracic Surgery, The Fourth Hospital of Hebei Medical University, Shijiazhuang, China

**Keywords:** Skip N2 lymph node metastasis, Stage III-N2 lung adenocarcinoma, Propensity score matching, Prognosis

## Abstract

**Purpose:**

Recent studies have indicated some differences in the prognosis of patients with stage III-N2 lung adenocarcinoma, and the prognosis of patients with skip N2 lymph node metastasis (SKN2) is good. This study grouped patients with stage III-N2 lung adenocarcinoma by propensity score matching (PSM) to evaluate the impact of SKN2 on the prognosis of these patients.

**Methods:**

The clinical data for patients who underwent radical lobectomy and had a postoperative pathological diagnosis of stage III-N2 lung adenocarcinoma at our centre from 2016 to 2018 were collected, and PSM was performed at a ratio of 1:1.

**Results:**

A total of 456 patients were enrolled in this study. After PSM, 112 patients were included in the SKN2 group, and 112 patients were included in the non-SKN2 group. When comparing the SKN2 group with the non-SKN2 group, the 3-year OS rate was (71.4% vs. 12.5%, p < 0.001), and the 3-year DFS rate was (35.7% vs. 5.4%, p < 0.001). It is further divided into four groups:single-station SKN2 (N2a1),Multi-station SKN2 (N2a2),single-station non-SKN2 (N2b1) and Multi-station non-SKN2 (N2b2).The 3-year OS and DFS rates of skip lymph node metastasis were better than those of non-skip lymph node metastasis(OS:N2a1 vs. N2b1 68.4% vs. 23.5%,p < 0.001;N2a2 vs. N2b2 73.0% vs. 7.7%,p < 0.001)(DFS:N2a1 vs. N2b1 68.4% vs. 5.9%,p < 0.001;N2a2 vs. N2b2 62.2% vs. 5.1%,p < 0.001), regardless of the number of N2 station(OS:N2a1 vs. N2a2 68.4% vs. 73.0%,p = 0.584;N2b1 vs. N2b2 23.5% vs. 7.7%,p = 0.051). On multivariate analysis, sex (p = 0.008) ,Vascular tumour thrombus(p = 0.047),size(p = 0.002)and SKN2 (p < 0.001) were independent predictors of OS.

**Conclusion:**

For patients with stage III-N2 lung adenocarcinoma, the prognosis of SKN2 patients is better than non-SKN2 patients’, and SKN2 may be used as an important factor in the N2 subgroup classification in future TNM staging.

## Introduction

Lung cancer has the highest incidence and is also the main cause of cancer-related deaths [1].Lymph node metastasis is one of the most important determinants of prognosis according to the TNM staging system [2].For approximately 20–40% of patients with lung adenocarcinoma, the postoperative pathological stage is III-N2, some studies have shown that some patients with lymph node metastases in N2 have a very poor prognosis; however, some specific patients with N2 disease have relatively good 5-year survival rates.

Recently, the International Association for Study of Lung Cancer (IASLC) proposed a new description of N, which combines the location of metastatic lymph nodes, N (single-station and multi-station) and skip N2 lymph node metastasis (SKN2) and further divides the N stage into multiple subgroups [2–5]. To date, these new N classifications have not been validated.Skip metastasis in mediastinal lymph node is defined as positive N2 metastasis with the absence of N1 lymph node metastasis in hilar and intrapulmonary lymph nodes. With about 17.2–42.3% of all patients with resected pN2-NSCLC [6, 7], the clinical significance of skip metastasis remains unclear. Many studies reported better prognosis in patients with skip metastasis compared with those with non-skip ones [8, 9].However, other works showed different outcomes [3].Because clinical pathological characteristics often differ between the two groups, the role of SKN2 in patients with stage III-N2 lung adenocarcinoma is difficult to clarify. The purpose of this study was to use propensity score matching (PSM) to reduce differences in clinical pathological characteristics and to further clarify the significance of SKN2 in the prognosis of patients with stage III-N2 lung adenocarcinoma.

## Methods

### Basic characteristics of the patients

The medical records of 456 patients who underwent surgery and had a postoperative pathologically confirmed diagnosis of stage III-N2 primary lung adenocarcinoma in the Department of Thoracic Surgery at our centre from January 2016 to December 2018 were reviewed. All patients underwent lobectomy and systematic lymph node dissection [10]. Lymph node stations were divided according to the lymph node map developed by the IASLC [11]. Systemic lymph node dissection was performed according to the recommendations of the European Association of Thoracic Surgeons (ESTS): at least 3 groups of mediastinal lymph nodes (including subcarinal lymph nodes) and hilar and intrapulmonary lymph nodes were included [12], and the number of lymph nodes removed was greater than 16 [13]. The SKN2 group included patients with lymph node metastasis at station N2 but without lymph node metastasis at station N1, and patients in the non-SKN2 group included patients with lymph node metastasis at stations N1 and N2. Patients with preoperative neoadjuvant chemotherapy or radiotherapy, non-R0 resection, and a non-adenocarcinoma pathology and without systemic lymph node dissection were excluded. These patients were staged according to the 8th edition of the TNM staging criteria.

### Postoperative adjuvant therapy

Patients with no contraindications for adjuvant therapy received postoperative platinum-containing dual-drug chemotherapy [10] or targeted therapy (if epidermal growth factor receptor (EGFR) gene-sensitive mutation was identified).

### Follow-up

The follow-up was performed every 3 months for the first 2 years after surgery and every 6 months over 2 years after surgery. The data update was based on the information obtained from telephone interviews, network interviews, and direct clinical visits. The deadline for follow-up was December 31, 2021.

### Statistical analysis

SPSS 22.0 statistical software (IBM, Armonk, NY) was used for statistical analysis. Patients’ age, sex, smoking history, visceral pleural invasion, vascular tumour thrombus, nerve invasion, EGFR gene mutation status, pathological type, pathological subtype, number of lymph node metastasis stations in N2 and tumour size were used for PSM at a ratio of 1:1, and the calliper used for matching was set to 0.1. Categorical variables were compared using Fisher’s exact test and the Pearson x^2^ test. Binary logistic regression analysis was used to determine the predictive factors of SKN2. The Kaplan-Meier method and log-rank test were used to compare overall survival (OS) and disease-free survival (DFS) curves. The Cox proportional hazards regression model was applied to perform univariate and multivariate analyses, and those variables that achieved statistical significance in univariate analysis were entered into the multivariable analysis.Kaplan-Meier survival analysis and the log-rank test were performed using R language, and survival curves were plotted.

## Results

A total of 456 patients were enrolled in this study, including 112 cases (24.6%) in the SKN2 group and 344 cases (75.4%) in the non-SKN2 group. Among the baseline characteristics of two groups of patients, sex (p = 0.002), EGFR gene mutation status (p < 0.001), and tumour size (p < 0.001) differed before PSM (Table 1).

**Table 1:** Baseline characteristics of the enrolled patients

Variable	Before PSM			After PSM		
	SKN2	Non-SKN2	*p*	SKN2	Non-SKN2	*p*
Age			0.358			0.073
< 65 y	76(67.9%)	249(72.4%)		76(67.9%)	72(64.3%)	
≥ 65 y	36(32.1%)	95(27.6%)		36(32.1%)	40(35.7%)	
Sex			0.002			0.681
Male	67(59.8%)	148(43.0%)		67(59.8%)	62(55.4%)	
Female	45(40.2%)	196(57.0%)		45(40.2%)	50(44.6%)	
Smoking history			0.894			0.383
Smoking	31(27.7%)	93(27.0%)		31(27.7%)	43(38.4%)	
No smoking	81(72.3%)	251(72.0%)		81(72.3%)	69(61.6%)	
Visceral pleura invasion			0.106			1.000
Invaded	36(32.1%)	140(40.7%)		36(32.1%)	39(34.8%)	
Not invaded	76(67.9%)	204(59.3%)		76(67.9%)	73(65.2%)	
Vascular tumour thrombus			0.280			0.067
Yes	21(18.8%)	88(25.6%)		21(18.8%)	29(25.9%)	
No	91(81.2%)	256(74.4%)		91(81.2%)	83(74.1%)	
Nerve invasion			0.065			0.811
Yes	9(8.0%)	51(14.8%)		9(8.0%)	13(11.6%)	
No	103(92.0%)	293(85.2%)		103(92.0%)	99(88.4%)	
EGFR mutation			< 0.001			0.811
Mutation	46(41.1%)	80(23.3%)		46(41.1%)	31(27.7%)	
No mutation	28(25.0%)	152(44.2%)		28(25.0%)	27(24.1%)	
No detection	38(33.9%)	112(32.6%)		38(33.9%)	54(48.2%)	
Size			< 0.001			0.410
≤ 3 cm	72(64.3%)	154(44.8%)		72(64.3%)	59(52.7%)	
> 3 cm	40(35.7%)	190(55.2%)		40(35.7%)	53(47.3%)	
Grade			0.071			0.221
Well differentiated*	71(63.4%)	249(72.4%)		71(63.4%)	62(55.4%)	
Poorly	41(36.6%)	95(27.6%)		41(36.6%)	50(44.6%)	
Metastasis stations			0.878			0.567
1 station	38(33.9%)	114(33.1%)		38(33.9%)	34(30.4%)	
> 1 station	74(66.1%)	230(66.9%)		74(66.1%)	78(69.6%)	

* comment: The highly differentiated types include acinar type, volt type and papillary type. The types of low differentiation include solid type and micropapillary type

**Fig. 1:**
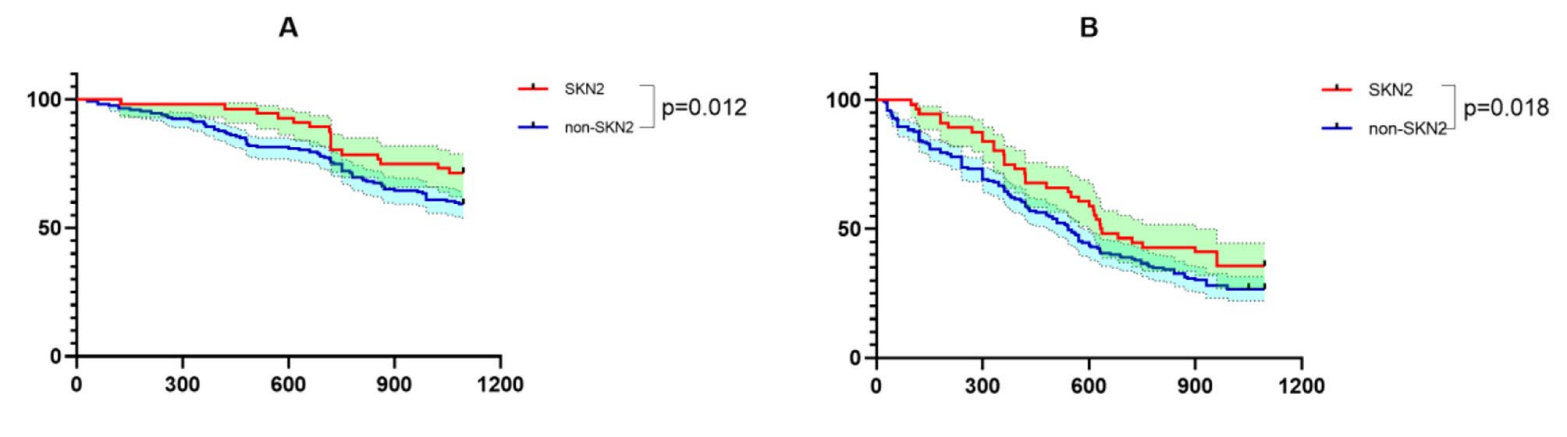
K-M survival curves of SKN2 and non-SKN2 patients before PSM Note: **A** is the OS curve; **B** is the DFS curve

In the entire cohort before PSM, the presence of SKN2 was significantly associated with better survival compared with the absence of SKN2 (OS, p = 0.012; DFS, p = 0.018), as shown in Fig. 1.

After PSM at a ratio of 1:1, 112 non-SKN2 patients and 112 SKN2 patients were included in the final analysis. The distribution of clinical parameters included for PSM is shown in Table 1. The univariate analysis using the Cox proportional hazards regression model indicated that the prognoses of male patients (p = 0.011), patients without vascular tumour thrombus (p = 0.007), patients without nerve invasion (P = 0.022), patients with EGFR mutation (p = 0.005), tumor size ≤ 3 cm(p = 0.003), and patients with SKN2 (p < 0.001) were relatively good. On multivariate analysis, male sex (p = 0.008), patients without vascular tumour thrombus (p = 0.047), tumor size ≤ 3 cm (p = 0.002) and SKN2 (p < 0.001) were independent prognostic factors (Table 2).

**Table 2:** Cox proportional hazard regression analysis

Variable	Univariate			Multivariate		
	Hazard ratio (HR)	95% confidence interval (CI)	*p*	HR	95%CI	*p*
Age						
< 65 y	0.932	0.645–1.347	0.708			
≥ 65 y	Reference					
Sex						
Male	0.638	0.452–0.901	0.011	0.621	0.437–0.882	0.008
Female	Reference			Reference		
Smoking history						
Smoking	1.169	0.816–1.676	0.394			
No smoking	Reference					
Visceral pleura invasion						
Invaded	Reference					
Not invaded	0.884	0.616–1.269	0.505			
Vascular tumour thrombus						
Yes	Reference			Reference		
No	0.585	0.396–0.863	0.007	0.670	0.451–0.995	0.047
Nerve invasion						
Yes	Reference					
No	0.549	0.329–0.916	0.022			
EGFR mutation						
Mutation	0.525	0.335–0.821	0.005	0.782	0.494–1.236	0.292
No mutation	Reference			Reference		
No detection						
Size						
≤ 3 cm	Reference			Reference		
> 3 cm	0.585	0.417–0.831	0.003	0.576	0.404–0.822	0.002
Grade						
Well	0.765	0.542–1.081	0.129			
Poorly	Reference					
Metastasis stations						
1 station	0.806	0.552–1.176	0.263			
> 1 station	Reference					
Skip N2 lymph node metastasis						
SKN2	5.785	3.855–8.681	< 0.001	5.525	3.657–8.345	< 0.001
non-SKN2	Reference			Reference		

**Fig. 2:**
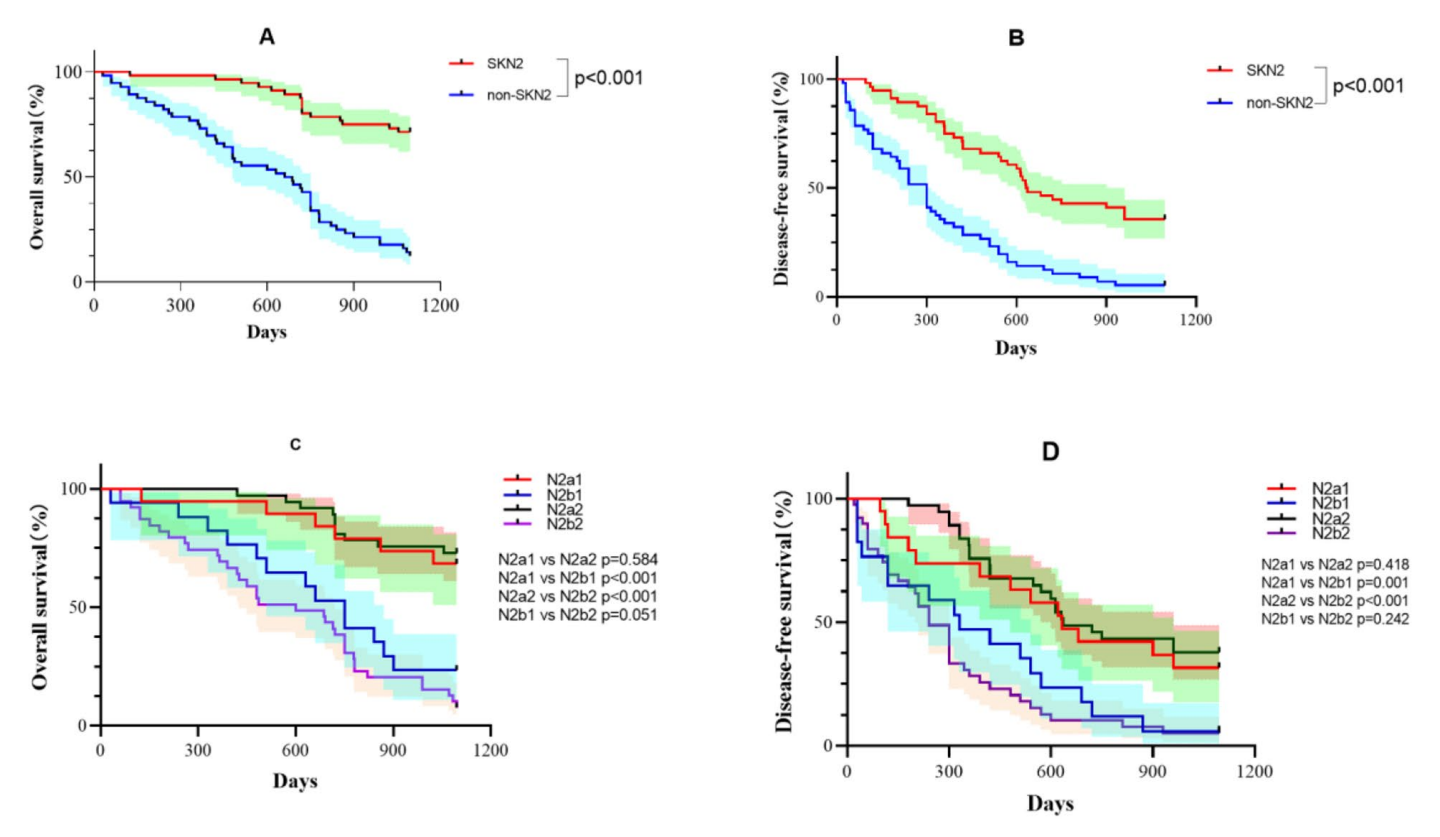
K-M survival curve after PSM Note: (**A**) K-M survival curves of the OS of the SKN2 and non-SKN2 groups, (**B**) K-M survival curves of the DFS of the SKN2 and non-SKN2 groups. (**C**) K-M survival curves of the OS of the four subgroups N2a1, N2a2, N2b1, and N2b2; (**D**) K-M survival curves of DFS of the four subgroups N2a1, N2a2, N2b1, and N2b2. N2a1: single-station SKN2, N2a2: multi-station SKN2, N2b1: single-station non-SKN2, N2b2: multi-station non-SKN2

After PSM, the baseline characteristics of the two groups were relatively matched. Figure 2 shows the OS curves of patients in the SKN2 group and the non-SKN2 group after PSM.The SKN2 group had a better 3-year OS rate (71.4% vs. 12.5%, p < 0.001) and a better 3-year DFS rate (35.7% vs. 5.4%, p < 0.001). The subgroup, as shown in Fig. 2,is further divided into four groups:single-station SKN2 (N2a1),Multi-station SKN2 (N2a2),single-station non-SKN2 (N2b1) and Multi-station non-SKN2 (N2b2).The 3-year OS and DFS rates of skip lymph node metastasis were better than those of non-skip lymph node metastasis(OS:N2a1 vs. N2b1 68.4% vs. 23.5%,p < 0.001;N2a2 vs. N2b2 73.0% vs. 7.7%,p < 0.001)(DFS:N2a1 vs. N2b1 68.4% vs. 5.9%,p < 0.001;N2a2 vs. N2b2 62.2% vs. 5.1%,p < 0.001), regardless of the number of N2 station(OS:N2a1 vs. N2a2 68.4% vs. 73.0%,p = 0.584;N2b1 vs. N2b2 23.5% vs. 7.7%,p = 0.051,DFS:N2a1 vs. N2a2 68.4% vs. 62.2%,p = 0.418;N2b1 vs. N2b2 5.9% vs. 5.1%,p = 0.242).

**Fig. 3:**
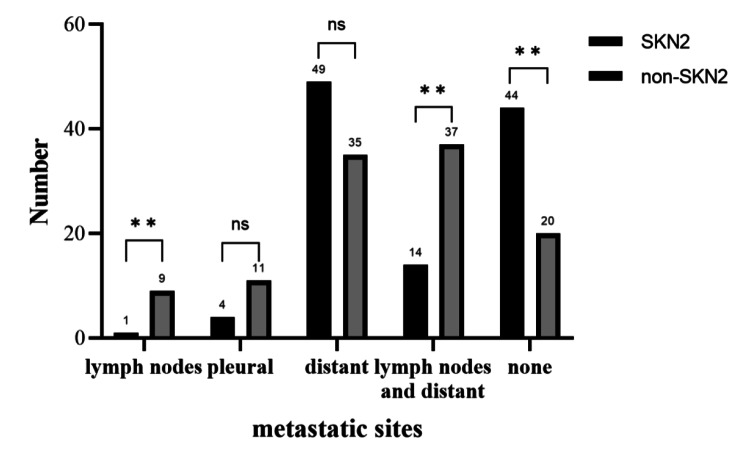
Summary of metastatic sites during the follow-up Note: ns corresponds to p > 0.05 between the two groups; ** corresponds to p < 0.01 between the two groups

In addition, the follow-up of this study indicated that the SKN2 group had a lower risk of metastasis than the non-SKN2 group, and the metastasis pattern was different, i.e., the SKN2 group had fewer lymph node metastases, more distant metastases, and relatively more recurrence-free patients (Fig. 3).

## Discussion

Lung cancer is the leading cause of cancer death. Because most cases are already in the late stage at the time of diagnosis, the prognosis is poor [1]. For patients with stage III-N2 lung adenocarcinoma after surgical treatment, the prognosis difference is found to be large during the follow-up. In other words, the former N2 lymph node classification may not meet the demand for accurate treatment of lung adenocarcinoma. How to more thoroughly classify pN2 has been a topic of research in recent years. When revising the 8th edition of the TNM classification for non-small cell lung cancer (NSCLC) [2–4], the IASLC divided pN2 into skip N2 single-station metastasis, non-skip N2 single-station metastasis, and N2 multi-station metastasis and proposes SKN2 as a basis for a new N2 subclassification. This paper differs slightly from the IASLC classification and divides N2 lymph nodes into four subgroups, namely, single-station SKN2 (N2a1), multi-station SKN2 (N2a2), single-station non-SKN2 (N2b1), and multi-station non-SKN2 (N2b2). Several previous studies have demonstrated that the prognosis of SKN2 patients is better than that of non-SKN2 patients [14]. However, in previous studies, significant baseline differences are usually evident between SKN2 group and non-SKN2 groups. Therefore, concluding that the survival rate of SKN2 patients is higher than that of non-SKN2 patients is difficult. In this study, PSM could maximally match the preoperative clinical baseline characteristic curves of the patients, thus providing more convincing data than previous papers.

Previous studies have focused on the clinical characteristics of SKN2 patients. The patient characteristics collected in this study indicated that males were more likely to develop SKN2 (p = 0.002), and other studies also reported the same conclusion. The studies of Wang and Xie reported that SKN2 was more common in patients with advanced ages (> 60 years), males, and patients who smoke [15, 16]. However, the data collected in this study did not indicate a significant difference between age (p = 0.358) and smoking history (p = 0.894). In addition, few studies have investigated the relationship between tumour size and SKN2. Stage IA lung cancer is known not to require follow-up treatment, and the tumour diameter is ≤ 3 cm. Wang et al. [15] reported that SKN2 was more common in tumours with a diameter ≤ 3 cm. This study found that the incidence of SKN2 was indeed higher for tumours ≤ 3 cm in diameter than for tumours larger than 3 cm. After PSM, tumour sizes were essentially consistent between the two groups (p = 0.410).

Considerable controversy remains regarding correlations between pathological types and SKN2. Most studies have shown no difference in the incidence of SKN2 in various pathological types of NSCLC. Some researchers believe that SKN2 is more likely to occur in squamous cell carcinoma [15, 17]. In this study, to avoid an influence of squamous cell carcinoma on the results, patients with adenocarcinoma were selected for this retrospective analysis.

A recent report indicated that [18] EGFR mutations were significantly more frequent in the SKN2 group (33%) than in the non-SKN2 group (10%) (p < 0.001). SKN2 and Kirsten rat sarcoma viral oncogene homolog (KRAS) mutations are not correlated. In this study, in the SKN2 group, 46 patients had EGFR mutations, 28 patients did not have EGFR mutations, and 38 patients did not undergo detection, with significant differences from the non-SKN2 group (p < 0.001). However, due to the high proportion of untested patients, this variable is only for reference.

Finally, most studies have demonstrated that SKN2 has a positive effect on OS [16, 19–21]. Tsitsias et al. [8] reported that the difference in OS between SKN2 patients and non-SKN2 patients was statistically significant (OS: 32.2 months vs. 24.2 months (p = 0.024)). A prospective study by Jiro et al. [9] indicated that the 5-year OS rates of the SKN2 and non-SKN2 groups were 81.3% and 37.5%, respectively, and the prognosis was significantly better in the SKN2 group. However, a few studies have reported opposite conclusions [22, 23]. Song [3] found that the survival of single-station SKN2 patients was not better than that of non-SKN2 patients, and the postoperative survival times of the patients in the two groups were similar (p = 0.93). However, these results should be rationally analysed, and the characteristics of nearly all of the patients enrolled in these studies differ. In the pathological data collected in this study, in male patients (p = 0.002), patients with EGFR mutations (p < 0.001), Single station lymph node metastasis (p = 0.017) and patients with a tumour diameter ≤ 3 cm (p < 0.001), SKN2 was prone to occur. Therefore, PSM was used to minimize the differences in patients’ pathological characteristics. In the post-PSM data, the clinical characteristics of the two groups were relatively similar, and 224 patients were enrolled after PSM. After 3 years of follow-up, when comparing the SKN2 group with the non-SKN2 group, the median OS rate was (no observed outcome vs. 673.5 days), the 3-year OS rate was (71.4% vs. 12.5%, p < 0.001), the median DFS was (634.5 days vs. 300 days), and the 3-year DFS rate was (35.7% vs. 5.4%, p < 0.001) (Fig. 2), suggesting that the SKN2 group was significantly better than the non-SKN2 group in terms of OS and DFS.

Further subgroup analysis of the study population (Fig. 2) indicated that after 3 years of follow-up, the 3-year OS and DFS rates of skip lymph node metastasis were better than those of non-skip lymph node metastasis(OS:N2a1 vs. N2b1 68.4% vs. 23.5%,p < 0.001;N2a2 vs. N2b2 73.0% vs. 7.7%,p < 0.001)(DFS:N2a1 vs. N2b1 68.4% vs. 5.9%,p < 0.001;N2a2 vs. N2b2 62.2% vs. 5.1%,p < 0.001), regardless of the number of N2 station(OS:N2a1 vs. N2a2 68.4% vs. 73.0%,p = 0.584;N2b1 vs. N2b2 23.5% vs. 7.7%,p = 0.051,DFS:N2a1 vs. N2a2 68.4% vs. 62.2%,p = 0.418;N2b1 vs. N2b2 5.9% vs. 5.1%,p = 0.242), suggesting that regardless of the number of lymph node stations with metastasis, the SKN2 group was better than the non-SKN2 group, which is different from the conclusions obtained by some studies. In a recent large-scale retrospective study, Yun et al. [5] reported that the prognosis of patients with multiple regional multiple lymph node metastases was worse than that of patients with single regional multiple lymph node metastases. The difference in this conclusion has many possible causes. First, PSM was initially performed to match the pathological characteristics of the two groups of patients in this study, which may have created some differences compared to the sample data used by Yun et al. Second, in this study, patients with multi-station N2 lymph node metastasis were further subdivided into multi-station N2 metastasis with or without N1 metastasis, and the results indicated that for patients with SKN2, regardless of single-station N2 or multi-station N2, both 3-year OS (68.4% vs. 73.0%, p = 0.584) and 3-year DFS (68.4% vs. 62.2%, p = 0.418) rates were not significantly different, while compared with the patients without SKN2, the difference was statistically significant regardless of the number of lymph node stations. Third, the sample size of the subgroup data in this study is small, and all data were from one centre.

Most of the results to date support the 8th edition of the TNM staging system of the American Joint Committee on Cancer, which uses the presence or absence of SKN2 as the condition for N2 subgroups. The new subclassification is the first landmark proposal, which clarifies the significance of the diversity of the N2 station and the existence of skip metastasis after surgery. The survival rate of patients with single-station SKN2 was significantly improved in N2-stage patients. The results of this study indicate that these patients should be carefully selected, and surgery may be the best treatment for these patients.

Mediastinal lymph node metastasis is a risk factor for postoperative recurrence and metastasis [24], especially for patients in the non-SKN2 group [25]. For stage III-N2 patients, chemotherapy or targeted therapy is inevitable after surgery, while the need for radiotherapy has not been determined [26]. Jin et al. [27] demonstrated that postoperative radiotherapy may be beneficial only for patients with single-station SKN2. Herskovic et al. [28] indicated that for stage IIIA-N2 NSCLC patients who received complete resection and multi-drug chemotherapy, postoperative radiotherapy could improve the prognosis. However, the follow-up of this study indicated that the SKN2 group had a lower risk of metastasis than the non-SKN2 group, and the metastasis pattern was different, i.e., the SKN2 group had fewer lymph node metastases, more distant metastases, and relatively more recurrence-free patients (Fig. 3), which can better explain why postoperative radiotherapy is beneficial for non-SKN2 patients but is ineffective for SKN2 patients and why the OS of the SKN2 group is better than that of the non-SKN2 group.

## Conclusion

In summary, SKN2 is an independent factor affecting the prognosis of patients with resectable stage III-N2 lung adenocarcinoma. In the current revision of the TNM system, N2 disease may be subdivided into more subgroups; therefore, new pN subgroup classifications should be proposed in the next TNM update.

## Data Availability

The datasets used and/or analyzed during the current study are available from the corresponding author on reasonable request.

## References

[1] Siegel RL, Miller KD, Fuchs HE, Jemal A (2022). Cancer statistics, 2022. CA Cancer J Clin.

[2] Asamura H, Chansky K, Crowley J (2015). The International Association for the study of Lung Cancer Lung Cancer Staging Project: proposals for the revision of the N descriptors in the Forthcoming 8th Edition of the TNM classification for Lung Cancer. J Thorac Oncol.

[3] Song H, Yoon SH, Kim J (2021). Application of N descriptors proposed by the International Association for the study of Lung Cancer in Clinical Staging. Radiology.

[4] Park BJ, Kim TH, Shin S (2019). Recommended change in the N descriptor proposed by the International Association for the study of Lung Cancer: a validation study. J Thorac Oncol.

[5] Yun JK, Lee GD, Choi S (2019). Comparison between lymph node station- and zone-based classification for the future revision of node descriptors proposed by the International Association for the study of Lung Cancer in surgically resected patients with non-small-cell lung cancer. Eur J Cardiothorac Surg.

[6] Riquet M, Assouad J, Bagan P (2005). Skip mediastinal lymph node metastasis and lung cancer: a particular N2 subgroup with a better prognosis. Ann Thorac Surg.

[7] Gorai A, Sakao Y, Kuroda H (2015). The clinicopathological features associated with skip N2 metastases in patients with clinical stage IA non-small-cell lung cancer. Eur J Cardiothorac Surg.

[8] Tsitsias T, Okiror L, Veres L (2021). New N1/N2 classification and lobe specific lymphatic drainage: impact on survival in patients with non-small cell lung cancer treated with surgery. Lung Cancer.

[9] Abe J, Matsumura Y, Shiono S (2020). Validation of the proposed cN2 subclassification in the Eighth Edition of the IASLC Staging System: a prospective phase II Multicenter Study. JTO Clin Res Rep.

[10] Kawasaki K, Sato Y, Suzuki Y, Saito H, Nomura Y, Yoshida Y (2015). Prognostic factors for surgically resected N2 non-small cell Lung Cancer. Ann Thorac Cardiovasc Surg.

[11] Rusch VW, Asamura H, Watanabe H, Giroux DJ, Rami-Porta R, Goldstraw P (2009). The IASLC lung cancer staging project: a proposal for a new international lymph node map in the forthcoming seventh edition of the TNM classification for lung cancer. J Thorac Oncol.

[12] Ma K, Chang D, He B (2008). Radical systematic mediastinal lymphadenectomy versus mediastinal lymph node sampling in patients with clinical stage IA and pathological stage T1 non-small cell lung cancer. J Cancer Res Clin Oncol.

[13] Liang W, He J, Shen Y (2017). Impact of examined Lymph Node count on Precise Staging and Long-Term Survival of Resected Non-Small-Cell Lung Cancer: a Population Study of the US SEER database and a chinese multi-institutional Registry. J Clin Oncol.

[14] Li H, Hu H, Wang R (2015). Lung adenocarcinoma: are skip N2 metastases different from non-skip. J Thorac Cardiovasc Surg.

[15] Wang L, Zhan C, Gu J (2019). Role of skip Mediastinal Lymph Node Metastasis for patients with resectable non-small-cell Lung Cancer: a propensity score matching analysis. Clin Lung Cancer.

[16] Li X, Li X, Fu X (2020). Survival benefit of skip metastases in surgically resected N2 non-small cell lung cancer: a multicenter observational study of a large cohort of the chinese patients. Eur J Surg Oncol.

[17] Chiappetta M, Lococo F, Leuzzi G (2020). External validation of the N descriptor in the proposed tumour-node-metastasis subclassification for lung cancer: the crucial role of histological type, number of resected nodes and adjuvant therapy. Eur J Cardiothorac Surg.

[18] Guerrera F, Renaud S, Tabbó F (2017). Epidermal growth factor receptor mutations are linked to skip N2 lymph node metastasis in resected non-small-cell lung cancer adenocarcinomas. Eur J Cardiothorac Surg.

[19] Wang X, Guo H, Hu Q, Ying Y, Chen B (2021). The impact of skip vs. non-skip N2 lymph node metastasis on the prognosis of Non-Small-Cell Lung Cancer: a systematic review and Meta-analysis. Front Surg.

[20] Wang Z, Cheng J, Huang W (2021). Skip metastasis in mediastinal lymph node is a favorable prognostic factor in N2 lung cancer patients: a meta-analysis. Ann Transl Med.

[21] Wang L, Ye G, Xue L (2020). Skip N2 metastasis in Pulmonary Adenocarcinoma: good prognosis similar to N1 disease. Clin Lung Cancer.

[22] Seyrek Y, Cansever L, Akın H, Metin M, Bolat E, Bedirhan MA (2021). The significance of skip Mediastinal Lymph Node Metastasis in the prognosis of patients with resected non-small-cell lung carcinoma: is it really a better N2 Disease Subtype. Ann Thorac Cardiovasc Surg.

[23] Zhao J, Li J, Li N, Gao S (2018). Clinical significance of skipping mediastinal lymph node metastasis in N2 non-small cell lung cancer. J Thorac Dis.

[24] Wen CT, Fu JY, Wu CF (2017). Risk factors for relapse of resectable pathologic N2 non small lung cancer and prediction model for time-to-progression. Biomed J.

[25] Isaka M, Kojima H, Takahashi S, Omae K, Ohde Y (2018). Risk factors for local recurrence after lobectomy and lymph node dissection in patients with non-small cell lung cancer: implications for adjuvant therapy. Lung Cancer.

[26] Nakagawa K, Yoshida Y, Yotsukura M, Watanabe SI (2021). Pattern of recurrence of pN2 non-small-cell lung cancer: should postoperative radiotherapy be reconsidered. Eur J Cardiothorac Surg.

[27] Jin J, Xu Y, Hu X (2020). Postoperative radiotherapy option based on mediastinal lymph node reclassification for patients with pN2 non-small-cell lung cancer. Curr Oncol.

[28] Herskovic A, Mauer E, Christos P, Nagar H. Role of Postoperative Radiotherapy in Pathologic Stage IIIA (N2) Non-Small Cell Lung Cancer in a Prospective Nationwide Oncology Outcomes Database. J Thorac Oncol. 2017. 12(2) 10.1016/j.jtho.2016.09.135 27746190

